# Supplementary material for: The genome sequence of a leaf beetle, Galeruca laticollis Sahlberg, C.R., 1838
Source: Wellcome Open Res. 2024 Oct 15;9:594. [Version 1] doi: 10.12688/wellcomeopenres.23195.1 (PMC11579587; doi:10.12688/wellcomeopenres.23195.1)
Supplement: Peer review history

# Reviewer report: Reviewer response for version 1

**DOI:** 10.21956/wellcomeopenres.25545.r109784

**Published:** 2024-11-20

**Authors:** Jose Manuel Rico Porras

**Affiliations:** 1 Universidad de Jaen, Andalusia, Spain

The article accurately describes the steps used for the assembly of the genome of the leaf beetle *Galeruca laticollis*. Some points to consider:

 You can include the family and order in the title: The genome sequence of a leaf beetle, *Galeruca laticollis *Sahlberg, C.R., 1838 (Coleoptera, Chrysomelidae)

 You can mention in the Background section that there are no cytogenetic data available for this species (Coleoptera Karyotype Database ^1^), but this data are available for some species of this genus ^2^:

*Galeruca angusta*: 2n=44M; 21+Xyp

*Galeruca* *artemisiae*: 2n=44M; 21+Xyp

*Galeruca* *canigouensis*: 2n=38-40M

*Galeruca* *indica*: 2n=46M; 22+Xyp

*Galeruca* *miegi*: 2n=38M; 18+Xyp

*Galeruca* *montícola*: 2n=38M; 18+Xyp

*Galeruca* *taceti*: 2n=40M; 19+Xyp

 In Table 2, Number of contigs is 643, but in the Genome assembly GCA_963921935.1 NCBI web is 642.

Are sufficient details of methods and materials provided to allow replication by others?

Yes

Is the rationale for creating the dataset(s) clearly described?

Yes

Are the datasets clearly presented in a useable and accessible format?

Yes

Are the protocols appropriate and is the work technically sound?

Yes

Reviewer Expertise:

Cytogenomics, repetitive DNA

I confirm that I have read this submission and believe that I have an appropriate level of expertise to confirm that it is of an acceptable scientific standard.

## References

[1] : Coleoptera Karyotype Database. The Coleopterists Bulletin. http://dx.doi.org/10.1649/0010-065X-69.1.174 10.1649/0010-065X-69.1.174

[2] : Chromosome Numbers and Meioformulae of Chrysomelidae. https://doi.org/10.1007/978-94-009-3105-3_10 10.1007/978-94-009-3105-3_10

# Reviewer report: Reviewer response for version 1

**DOI:** 10.21956/wellcomeopenres.25545.r109779

**Published:** 2024-11-20

**Authors:** Željko Tomanović

**Affiliations:** 1 Serbia University of Belgrade, Belgrade, Serbia

The authors presented the sequenced genome of the chrysomelid beetle Galeruca laticollis using a male specimen collected in Wheatfen Broad, England, United Kingdom. The authors gave a brief overview of the biology of G. laticollis. All relevant quantitative parameters of the assembled genome are listed and the methodology presented in detail. The following is just a suggestion.

 "Outside the UK, it occurs in most of Europe and extends eastwards into temperate parts of Asia."

 Please add appropriate references on the distribution of the species.

Are sufficient details of methods and materials provided to allow replication by others?

Yes

Is the rationale for creating the dataset(s) clearly described?

Yes

Are the datasets clearly presented in a useable and accessible format?

Yes

Are the protocols appropriate and is the work technically sound?

Yes

Reviewer Expertise:

phylogenetics and biodiversity

I confirm that I have read this submission and believe that I have an appropriate level of expertise to confirm that it is of an acceptable scientific standard.
